# Charles Dickens' Hypnagogia, Dreams, and Creativity

**DOI:** 10.3389/fpsyg.2021.700882

**Published:** 2021-07-27

**Authors:** Marleide da Mota Gomes, Antonio E. Nardi

**Affiliations:** ^1^Laboratory of History of Psychiatry, Neurology, and Mental Health, Institute of Psychiatry, Institute of Neurology, Federal University of Rio de Janeiro, Rio de Janeiro, Brazil; ^2^Laboratory of History of Psychiatry, Neurology, and Mental Health, Institute of Psychiatry, Federal University of Rio de Janeiro, Brazilian Academy of Science, National Academy of Medicine, Rio de Janeiro, Brazil

**Keywords:** dream, lucid dream, REM sleep, hypnagogia, creativity

## Introduction

Charles Dickens was a prolific and ingenious 19th-century English writer who died just over 150 years ago, in 1870. He was also an insomniac versed in sleep disorders (Cosnett, 1992; Collins, 2020). Dickens was a social reformer and supported the development of shelters for homeless women, the United Kingdom's first pediatric hospital, and humane treatment of the underprivileged (Kryger, 2012).

As we will show, the virtual dream world was central to Dickens' narrative structure and was fundamental to his work process, social engagement, and fantasy, as we can also infer from Winyard and Furneaux (2010): “For Dickens, fundamental emotional and social bonds are formed between different classes and peoples by creating sympathy through *imagination*,” and “For Dickens, science should excite, rather than reductively explain*, imagination* and the bonds that forge it between people.”

Another issue in Dickens's life was hypnagogia, referring *lato sensu* to fleeting perceptual experiences during the transition from wakefulness to sleep, with the common occurrence of involuntary and imagined experiences, hypnagogic hallucinations, and from sleep to wakefulness, hypnopompic hallucinations (Waters et al., 2016). Dreams themselves are expressed by varying degrees of emotional intensity, bizarreness, visual vivacity, and narrative complexity, but without metacognition (Yu and Shen, 2020; Scarpelli et al., 2021).

The hypnagogic state is useful for problem-solving and creative work. REM sleep and dreams are linked even more to creativity (Llewellyn and Desseilles, 2017). This association was reinforced by the Franco-Italian study of patients with narcolepsy, characterized by falling asleep directly in REM sleep and, among several symptoms, a high frequency of dream recall and also lucid dreams, when a person becomes aware that he is dreaming while remaining physiologically asleep and immersed in a dream (Yu and Shen, 2020; Mota-Rolim et al., 2021). These results highlight a greater creative potential in these individuals and further support the role of REM sleep in creativity (Lacaux et al., 2019). According to studies, creative insight depends on the spread of neural activation to make remote associations between memories (Llewellyn and Desseilles, 2017).

Importantly, hypnagogia, lucid dreaming, and dissociative thinking during dreaming have been reported for thousands of years in various religions and by many philosophers (Mota-Rolim et al., 2021).

This article aims to explore hypnagogia and dreams in two of Dickens' most famous characters, Oliver Twist in *Oliver Twist* (1837-1839) (Dickens, 1838, 1839a,b) and Ebenezer Scrooge in *A Christmas Carol* (Dickens, 1843). This is consistent with some literary critics who have focused their attention on Dickensian sleep/dreams. However, the biological approach to his work, which is the main objective of this article, is unusual.

## Manuscript Formatting

### Charles Dickens' Dream World

Dickens' traumatic experiences as a teenager, as well as his wanderings in the streets of London, served as raw material for his creative process, as well as for the composition of his characters. He also portrayed vivid dreams like those of Ebenezer Scrooge, as well as sleep disorders, possibly drawing on his own experiences and those of his family and friends (Dickens, 1843; Cosnett, 1992).

Dickens described sleep problems in his characters, including insomnia; sleep promotion, places that favor sleep, soporific effects of meals; hypnagogic hallucinations; perhaps the first report of restless legs syndrome, sleep paralysis; dreams, nightmares, and night terrors; somnambulism and drowsiness (Cosnett, 1992). The latter symptom appears in a boy, Joe, a character in one of his books, who was obese, very sleepy during the day and prone to snoring. According to Cosnett (1992), Dickens must have realized the potential severity of this syndrome. Impressive! Charles Dickens was one of the first informal experts in sleep and wake disorders, many of which had not yet been characterized in his time.

**Figure 1:**
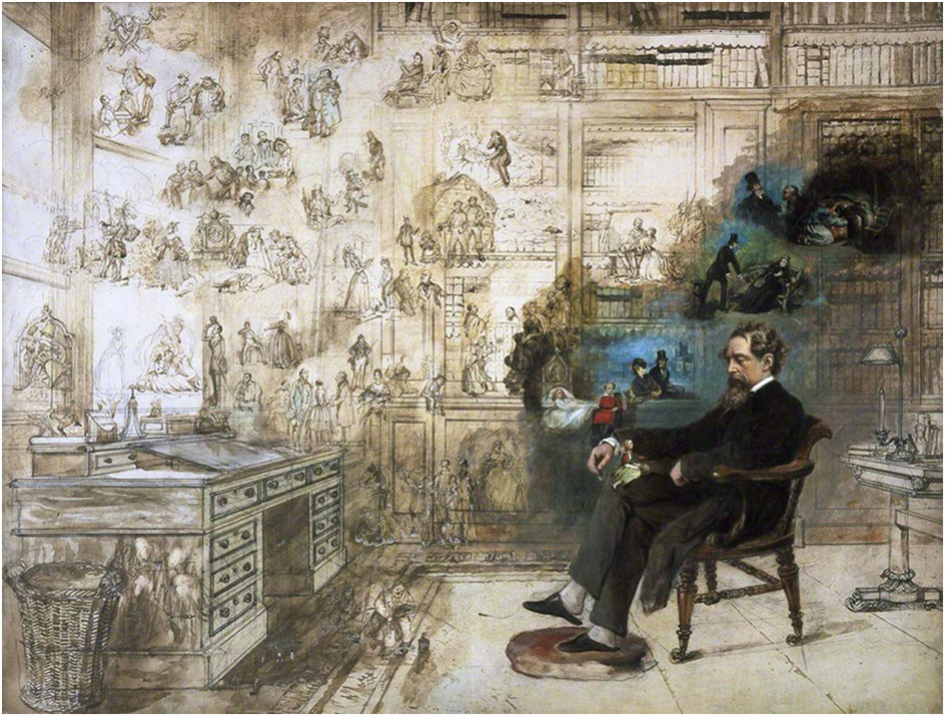
Dickens' Dream (1875) watercolor painting by Robert William Buss about Dickens and his vivid mind with characters from all his books, while he sleeps in his library at Gad's Hill. Picture: Public domain.

The famous writer is known for having used his dreams (Figure 1) as “aesthetic experiences of intrinsic value” for his creative fiction (Chowdhury, 2014). He grew up under the influence of the Romantic Movement and its writers, who identified a strong connection between dreams and the process of literary creation (Chowdhury, 2014).

Dickens recognized the importance of sleep in his own creative mind, as well as in his contemporaries. The current study discusses some accounts of Dickens' characters in light of present-day neurobiology and the creativity of dreams.

Dickens suffered from insomnia. To control his affliction, among other measures, he would get out of bed and walk the streets of London so that he could enjoy restful sleep upon his return (Chowdhury, 2014). During these walks he pictured useful scenes for his writings and transformed his empathy for the suffering of others into a medium for social reform.

The writer also had the habit of lying in the middle of his bed, facing north, his arms outstretched and his hands equidistant from the edges. He kept a compass to ensure that he slept facing north. Dickens also believed that this particular practice enhanced his creativity (Horne, 2016). Sleep-inducing substances such as alcohol and opium or laudanum tincture were widely available in 19th-century England and were thus available to anyone who could afford them, including Dickens.

In the course of his career, Dickens published novels with numerous memorable “Dickensian” characters. *Oliver Twist* (1837-1839), Dickens' second novel, tells the life of an orphan who lives on the streets and his first nine years in charity shelters. Unable to endure so much mistreatment, Oliver flees to London, where he joins a group of criminals led by Fagin. The boy experiences much suffering before finally living happily with an inheritance left by his father and an unexpected family.

*A Christmas Carol* (Dickens, 1843) features Ebenezer Scrooge, a miser who catches a glimpse of the ghost of his late business partner, and who regrets being neither good nor generous. According to the ghost, Scrooge will receive a vision of three more ghosts, who will take him on a journey through the present, past, and future, to try to save him while he is still alive. All four ghosts visit him in a single night, but Scrooge is unsure whether they are a dream or reality.

### Oliver Twist's and Ebenezer Scrooge's Intriguing Scenes Vis-à-Vis Creative Thinking and Dreams

Before this specific assessment, we should note that sleep has a specific architecture, described as a cyclical occurrence of rapid eye movement (REM) sleep and non-REM sleep (stages N1, N2, and N3) (Iber et al., 2007).

In addition, dramatic changes in brain neurobiology and dream states of consciousness distinguish the different stages of sleep from one another, suggesting specific roles for memory and psychological processes (Kirov and Brand, 2012).

Brain research has shown that lucid dreaming and metacognition share similar neural systems (Yu and Shen, 2020; Mota-Rolim et al., 2021), but that with different levels of metacognition, the weirdness of lucid dreaming also changes (Yu and Shen, 2020). Besides, dreams' bizarre strangeness is considered a crucial feature of their content and can be seen as the result of impaired cognitive processing (Hobson et al., 2000; Yu and Shen, 2020).

It is already understood that the brain generates predictions (or expectations) to explain and thus recognize sensory input based on past experience. More recent hypotheses point to the role of the predictive brain during altered states of consciousness, suggesting REM sleep's special participation. As stated by Llewellyn, 2016: “The REM dream constitutes a form of prospective image-based code which identifies an associative pattern in past events and, therefore, portrays associations *between* past experiences (rather than the experiences as such). This image-based code may be retained at an unconscious level and mobilized to predict the immediate sensory environment and interpret the causes of sensory input during wake” (Llewellyn, 2016). Consequently, according to this theory, the REM dream could represent a virtual reality when the brain generates predictions to interpret sensory input by identifying a probabilistic pattern of past events.

Furthermore, in healthy individuals, dreaming can favor memory consolidation, while in those with post-traumatic stress disorder or nightmares, as well as in “stressed” individuals, there is a new virtual reality, when daytime traumatic content is incorporated into problem-solving strategies (Scarpelli et al., 2019).

Various dream scenes experienced by Dickens' characters have often been examined from a psychological or psychodynamic point of view. Here, we attempt to interpret such scenes from a neurobiological point of view, while not dismissing the importance of psychological traumas in the expression of oneirism.

Current neurobiological knowledge supports the two main hypotheses on the production, elaboration, and remembering of the dream experience, namely the continuity hypothesis and the activation-synthesis hypothesis. The continuity hypothesis was proposed in the early 1970s and suggests that the content of dreams is continuous with the dreamer's concepts and concerns upon waking up. This hypothesis includes the concepts of daytime residue effect (a reflection of events in dreams one or two nights after their occurrence), depending on the vivid emotional context (Veloce et al., 2019). Or, the effect of delaying the dream, that is, the reappearance of daytime events ~one week later, is more likely when personally significant events are encountered (Veloce et al., 2019).

The activation-synthesis hypothesis was proposed by John Allan Hobson and Robert McCarley (1977) with a neurobiological basis of dreams related to differences in brainstem neuronal activity during wakefulness and REM sleep. According to the hypothesis, dreams result from activation of the brain during REM sleep. The theory was improved as the technology and experimental equipment become more accurate. This was an anti-Freudian theory, a highly influential neuroscientific account of dreaming that rejected the notion of dreams originating from a “meaning” subject to decoding (Hobson et al., 2000; Wamsley and Stickgold, 2010).

When the images are vivid or disturbing, hypnagogic hallucinations as highly realistic visions are encountered most often in narcolepsy. According to the activation-synthesis hypothesis, these hallucinations result from REM-like enhancement of internal stimuli coupled with an activated, aminergically modulated waking brain (Hobson et al., 2000).

According to the activation-synthesis model, the dream is experienced when the sleeping brain attempts to make sense of the chaotic entry at its upper cortical level, which can happen in both REM and non-REM sleep, sometimes as vivid, bizarre, and similar to a story as the REM dream. Today, the dream experience is seen as one of several forms of spontaneous offline cognition that involves reactivating and processing memory during resting states (Wamsley and Stickgold, 2010). Besides, chaotic neural states during dreaming may be closely related to dissociative states that manifest themselves in the REM sleep dream scenario. Thus, the underlying neural processes that allow a new integration of contents dissociated from memory can generate new ideas, insights, and other creative states of consciousness (Bob and Louchakova, 2015).

**Figure 2:**
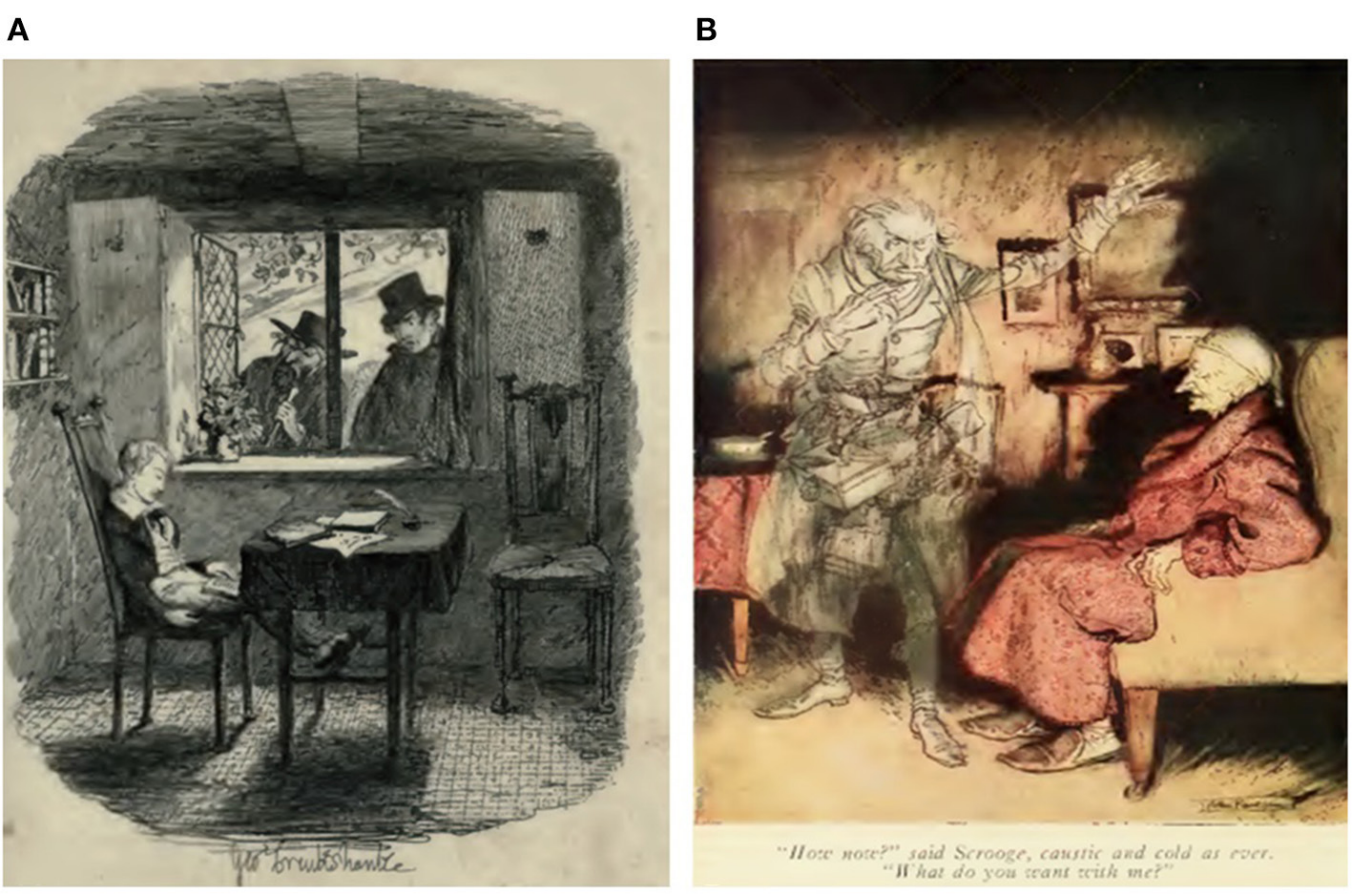
The dreamlike mental state experienced in two Dickens' characters. **(A)**. Oliver saw Fagin and Monks through the windows (*Oliver Twist*, v.2, p. 261). **(B)**. Ebenezer Scrooge is visited by the ghost of his former business partner Jacob Marley (*A Christmas Carol*). Drawings: public domain.

When Oliver Twist falls asleep in the sitting position, he sees his enemies, Monks and Fagin, apparently in an episode of dream-reality confusion, from the transition between waking and superficial sleep (Figure 2A). This may be due to the day-residue effect, or, better yet, the dream-lag effect, as happened with Oliver (Veloce et al., 2019). Consequently, both effects are pertinent in Oliver Twist's memories of being chased by the two delinquents, Monks and Fagin.

However, moderate-to-severe interpenetration of the waking, sleeping, and dreaming phases can precipitate psychopathologies, since the distinct functions of these phases are partially impaired. For example, in attention-deficit hyperactivity disorder, these phases are undifferentiated or labile (Llewellyn and Desseilles, 2017). Consequently, psychopathologies can appear if the dreaming and waking states become hybridized, as demonstrated by Skrzypińska and Szmigielska (2015) in patients with borderline personality disorder and by van Heugten-van der Kloet and Lynn, 2020 in patients with dissociation.

There is no doubt that Oliver Twist faces social and psychological problems, and these associations must be considered (van Heugten-van der Kloet and Lynn, 2020). The characters displays aspects of dissociation of psychobiological functioning, especially impacting consciousness and perception, as well as hybridization of levels of consciousness. This happens in Oliver's possible generalized anxiety disorder (GAD), since he has difficulty controlling his brooding and worrying. The Diagnostic and Statistical Manual of Mental Disorders, 5th Edition (DSM-5), lists among the criteria for GAD: extreme anxiety, worrying, and difficulty sleeping (due to trouble falling asleep or staying asleep, restlessness at night, or unsatisfactory sleep) (American Psychiatric Association, 2013).

Dissociative symptoms can potentially disrupt all areas of psychological functioning, including awareness, memory, identity, emotion, perception, body representation, motor control, and behavior (DSM-5). However, these symptoms are not limited to dissociative disorders *per se*, as they can also reflect a trans-diagnostic and comorbid phenomenon with psychotic illnesses and anxiety and depression, for example. The intensity of these symptoms is believed to relate to the severity of hybridity.

Some literary critics have focused their attention on Dickensian sleep. For example, Andrade (*apud* Greaney, 2014) shows that oscillation between sleeping and waking states are crucial to the novel's narrative structure and notes that “Oliver undergoes too many levels of unconsciousness, semi-consciousness, and consciousness, for the reader to be able to distinguish one from the other.” This hybridization is clearly stated in Oliver Twist: “…reality and imagination become so strangely blended that it is afterwards almost a matter of impossibility to separate the two…” (Dickens, 1839a in *Oliver Twist*, 259).

Furthermore, there are also several accounts of dream-reality confusion when for example Scrooge is confused about his first dream (Dickens, 1843, p. 39):

“He resolved to lie awake until the hour was passed; and, considering that he could no more go to sleep than go to heaven, this was, perhaps, the wisest resolution in his power. The quarter was so long, that he was more than once convinced he must have sunk into a doze unconsciously, and missed the clock.”

This next excerpt also sounds like an account of a hypnagogic state, but it could also be a lucid dream in REM sleep (Dickens, 1838 in Oliver Twist, p. 134–135):

“There is a drowsy state, between sleeping and waking, when you dream more in five min with your eyes half open, and yourself half conscious of everything that is passing around you than you would in five nights with your eyes fast closed, and your senses wrapt in perfect unconsciousness.”

The state of hypnagogic sleep is similar to that of REM sleep. Hypnagogia is a common fleeting perceptual experience that occurs during the transition from wakefulness to sleep and from sleep to wakefulness, with varying degrees of emotionality (Waters et al., 2016). Concerning lucid dreams, one is dreaming during sleep, with most dreams occurring during REM but also during non-REM sleep.

This excerpt depicts the common dreamlike state of Dickens and his characters (Dickens, 1838 in *Oliver Twist*, p. 115):

“I hope so,’ replied the child,‘after I am dead, but not before. I know the doctor must be right, Oliver, because I dream so much of heaven and angels, and kind faces that I never see when I am awake.’ ‘Kiss me,’ said the child, climbing up the low gate, and flinging his little arms round Olivers neck. ‘Goodbye, dear God bless you!”

The next excerpt sounds like confusional arousals that are more common in children and that happen from partial or incomplete arousal from a deep sleep: “Weak, and thin, and pallid, he awoke at last from what seemed to have been a long and troubled dream” (Dickens, 1838 in Oliver Twist, p. 176).

This excerpt, in the context of the chapter, appears to be just a description of a drowsy child reacting to an affectionate gesture (Dickens, 1839a in Oliver Twist, p. 164–165): “The boy stirred, and smiled in his sleep, as though these marks of pity and compassion had awakened some pleasant dream of a love and affection he had never known.”

Regarding Ebenezer Scrooge (Figure 2B), it appears that his dream content exhibits oscillatory changes throughout the night, from short reports to elaborate, vivid, emotional dreams with sensorimotor hallucinatory experiences that are common in REM-sleep dreams (Carr and Solomonova, 2019). As noted above, dream-like mental activity can be observed during all phases of sleep. However, in REM sleep, dreams tend to be more vivid, emotional, bizarre, and more often include an associated narrative structure as suggested with the patterns of neural activation and deactivation observed during REM sleep. For example, during this dream state, on functional neuroimaging scans, visual areas appear more active, compared to both wakefulness and slow-wave sleep. In summary, dream states, especially those of REM sleep, are characterized by high activity in areas of the brain associated with imagery. This phase is also marked by reduced activity in the prefrontal cerebral cortex involved in planning, decision-making, and social behavior.

The volume of the left amygdala is related to dreams' bizarreness, but a smaller volume of the left hippocampus and a larger volume of the right hippocampus were related to emotional load, as reported by Scarpelli et al. (2021).

The main character in *A Christmas Carol* apparently experienced a dream triggered in the sleep-onset REM period (SOREMP), with suggestive descriptions caused by his fatigue (Dickens, 1843, p. 34, 65):

“And being, from the emotions he had undergone, or the fatigues of the day, or his glimpse of the Invisible World, or the dull conversation of the Ghost, or the lateness of the hour, much in need of repose, went straight to bed without undressing, and fell asleep upon the instant.”

“He was conscious of being exhausted, and overcome by an irresistible drowsiness, and, further, of being in his own bedroom. He gave the cap a parting squeeze, in which his hand relaxed; and had barely time to reel to bed, before he sank into a heavy sleep.”

Dreaming can range from mentation that is typical of the early stages of non-REM sleep to vivid dreams that are more typical of REM sleep. In this stage, extensive sensorimotor cortical activity appears to underlie the vividly embodied images of dreaming, as commented above. One theory is that the dream is influenced by muscular twitching, as also occurs during early sleep, when the experience of hypnotic jerks can be associated with vivid images of falling. In addition to muscular twitches, Carr et al. (2020) claim that cortical processing of body sensation continues during sleep and influences dream generation. Dreaming thus emerges in a co-creative way: actual body sensation contributes to dreaming generation just as individual experience shapes the dream narrative, which manifests in more bodily sensation, and so on, completing the dreaming circuity (Carr et al., 2020).

Scarpelli et al. (2019) raised questions in their literature review on disturbing dreams in life. The authors assume that dreams can be considered an expression of brain maturation and cognitive development, consequently concerning memory and visuospatial abilities. Furthermore, the mind-sleep activity could be beneficial in the case of stressful events. However, there are differences in the experiences of both sleep and dreams throughout life. Older adults tend to remember the dream contents related to essential autobiographical memories, as was the case with Scrooge, the old miser. However, there is a dramatic decline in the rate of dream recall when brain damage occurs. In particular, bad dreams and nightmares can attempt to deal with stressful events at any age, but periods characterized by changes in childhood seem to be more related to disturbing dreams. This is established in Oliver Twist's experiences of floating consciousness. However, older adults affected by anxiety symptoms during wakefulness report higher rates of nightmares, but reduced dreaming has been observed during aging.

While dreaming in healthy persons may serve memory consolidation processes, in post-traumatic stress disorder or in “stressed” patients, it could promote the simulation of a new reality, where traumatic daytime contents are modified or integrated into strategies to aid problem-solving mechanisms. These latter considerations may be related to Oliver's experiences with his persistent persecution by the delinquents led by Fagin.

### Conclusions and Emerging Ideas

Charles Dickens outlined a variety of manifestations of sleep and its disturbances, presumably related to his own experience and that of family and friends, also expressed in some of his characters.

There are many reports of the dream-reality confusion through the anguished young Oliver Twist, the protagonist of the book by the same name. The virtual world of dreams is important for Dickens' narrative structure, especially in the experiences of Oliver Twist, as well as in the lively and extensive perceptions (with numerous scenes) of Ebenezer Scrooge, an old miser, in *A Christmas Carol*.

Dreams thus fill the gap between sleep and conscious cognition. They are oneiric conceptions that are reported in both young and old. Regional activation of neural networks involved in memory and imagination can play a role in the process by which memories combine in fictitious scenarios during the dream, so dreaming and waking cognition share a common neurobiological substrate (Graveline and Wamsley, 2015).

Dickens apparently used sleep, usually hypnagogia, perhaps REM lucid dream, as a creative work process, as seen more easily in narcoleptics. He portrayed these phenomena in his characters. The current study refers to some reports of Dickens' characters in light of our present-day understanding of sleep and dream neurobiology.

Fluctuating cognitive activity is presented by Charles Dickens through dreaming by his young or old characters. Dreams, especially those in non-REM and hypnagogia, in the transition from non-REM sleep to wakefulness, can be expressed through altered perceptions in a fluctuating state of consciousness (Waters et al., 2016). The content of thought and images during waking at rest also shares many characteristics with the dream experience. However, daydream reports are typically shorter and less fantastic than REM sleep dreams, which can be visually vivid and even bizarre (Wamsley and Stickgold, 2010).

This happens because dreams favor creativity and problem-solving. In the transition from sleep to wakefulness, the mind is free to make creative associations and assimilate information without the usual critical scrutiny of the waking state.

Some of Charles Dickens' characters confuse reality with imagination when non-real apperceptions seem falsely familiar, as is the case of scenes with young Oliver Twist and old Ebenezer Scrooge. There is also the character with vivid accounts of dreams with a long and complex autobiographical plot, as is the case with Scrooge, the old miser who experiences a process of redemption at Christmas time.

In addition to his literary work *per se*, Charles Dickens left a legacy on the use of dreaming as a means for generating creative insights, now better understood through the neurobiology of dreams.

## Author Contributions

All authors gave substantial contributions to the conception of the work MM made the acquisition of the references, the first drafting of the work, and AN revised it critically for important intellectual content the final approval of the version to be published are given by all authors.

## Conflict of Interest

The authors declare that the research was conducted in the absence of any commercial or financial relationships that could be construed as a potential conflict of interest.

## Publisher's Note

All claims expressed in this article are solely those of the authors and do not necessarily represent those of their affiliated organizations, or those of the publisher, the editors and the reviewers. Any product that may be evaluated in this article, or claim that may be made by its manufacturer, is not guaranteed or endorsed by the publisher.
